# Microbleeds in dementia with Lewy bodies

**DOI:** 10.1007/s00415-020-09736-0

**Published:** 2020-02-04

**Authors:** Paul C. Donaghy, Michael Firbank, Dipayan Mitra, George Petrides, Jim Lloyd, Nicola Barnett, Kirsty Olsen, Alan J. Thomas, John T. O’Brien

**Affiliations:** 1 Translational and Clinical Research Institute, Newcastle University, Newcastle upon Tyne, UK; 2 Neuroradiology Department, Newcastle Upon Tyne Hospitals NHS Foundation Trust, Newcastle upon Tyne, UK; 3 Nuclear Medicine Department, Newcastle Upon Tyne Hospitals NHS Foundation Trust, Newcastle upon Tyne, UK; 4 Department of Psychiatry, University of Cambridge, Cambridge, UK

**Keywords:** Dementia with lewy bodies, Microbleeds, Imaging, Amyloid, Vascular

## Abstract

**Introduction:**

Microbleeds are associated with the development of dementia in older people and are common in Alzheimer’s disease (AD). Their prevalence and clinical importance in dementia with Lewy bodies (DLB) is unclear. The objective of this study was to compare the rates of microbleeds in DLB with those in AD and healthy older people, and investigate associations between microbleeds and amyloid deposition, vascular risk and disease severity in DLB.

**Methods:**

DLB (*n* = 30), AD (*n* = 18) and control (*n* = 20) participants underwent clinical assessment at baseline and 1 year in this longitudinal observational study. 3T MRI (including T2* susceptibility weighted imaging) and florbetapir PET were carried out at baseline. Microbleeds were rated visually and a standardised uptake value ratio (SUVR) was calculated from florbetapir PET scans.

**Results:**

40% of DLB subjects had microbleeds compared with 50% of AD and 15% of controls. Compared to DLB without microbleeds, those with microbleeds had higher systolic BP (156 ± 26 v. 135 ± 19 mmHg; *p* = 0.03), but did not have greater levels of vascular disease or amyloid deposition (SUVR 1.25 ± 0.24 v. 1.25 ± 0.22; *p* = 0.33). There was evidence of less severe dementia in DLB participants with microbleeds, but these differences may have been driven by a shorter disease duration in those with microbleeds.

**Conclusion:**

The presence of microbleeds in DLB is associated with higher blood pressure, but not with other measures of vascular disease or amyloid deposition. The relationship between microbleeds and clinical presentation remains unclear.

**Electronic supplementary material:**

The online version of this article (10.1007/s00415-020-09736-0) contains supplementary material, which is available to authorized users.

## Introduction

Dementia with Lewy bodies is characterised by alpha-synuclein (αSyn) deposition in brainstem, limbic and cortical areas [1]. This is often accompanied by other pathologies such as amyloid [2], tau [3] and vascular pathology [4]. Certain types of vascular pathology, such as microinfarcts, atherosclerosis and cerebral amyloid angiopathy, are more common in synucleinopathy than in healthy controls [5]. In addition, white matter changes are more widespread in DLB than controls both on MRI and postmortem [6, 7], though these changes do not necessarily reflect vascular disease [8]. Infarcts on MRI and larger infarcts on postmortem do not appear to be more common in DLB than controls [5, 6]. The presence of vascular disease may have an additive effect on cognitive impairment in neurodegenerative diseases, as vascular disease has been shown to be an independent predictor of dementia in both Alzheimer’s disease and synucleinopathy [5].

Cerebral microbleeds are small foci of chronic blood products in normal or near normal brain tissue [9]. They appear as small hypointense areas on T2*-weighted MRI due to the presence of superparamagnetic haemosiderin. Microbleeds can be classed as lobar (cortical or subcortical), deep (basal ganglia, thalamus, internal capsule, external capsule, corpus callosum or deep/periventricular white matter) or infratentorial (brainstem or cerebellum) [10]. Lobar microbleeds have also been shown to be associated with cortical amyloid deposition on PET amyloid imaging and the APOE ε4 allele, whereas deep microbleeds are associated with cardiovascular risk factors [11–13].

The effect of microbleeds on cognition in dementia is unclear. Microbleeds are more common in AD than in healthy older people (odds ratio 2.1), but are not associated with differences in global cognition within AD [14]. The actual prevalence of microbleeds is difficult to ascertain as their detection increases with increased MR scanner field strength and the use of more sensitive imaging techniques such as susceptibility weighted imaging [14].

Microbleeds have been reported as being present in 17–45% of cases of DLB [15–17]. Two studies have reported that microbleeds are present in similar proportions of DLB and AD cases [16, 17]. One study reported that microbleeds were associated with a trend towards less reduction in cardiac MIBG binding, suggesting that vascular pathology was associated with less severe Lewy body pathology in DLB [16]. The distribution of microbleeds in DLB has differed between studies, though frontal microbleeds appear to be common. In contrast to findings in AD, a postmortem study in Lewy body dementia found that microbleeds were not more common in cases with AD pathology or cerebral amyloid angiopathy [18]. The clinical relevance of microbleeds in DLB is unclear.

### Aims and hypotheses

The aim of this study was to compare the rates of microbleeds in DLB with those in AD and healthy older people, and to compare the clinical and imaging findings of DLB with and without microbleeds. The hypotheses were that:

1. Microbleeds would be more common in DLB than healthy controls, at a rate similar to AD.
2. The presence of microbleeds in DLB would not be associated with amyloid deposition.
3. DLB with microbleeds would be associated with greater dementia severity and more rapid progression over 1 year.

## Methods

### Participants

Participants with dementia were recruited prospectively between June 2013 and February 2016 from secondary care services in the North of England. All participants were ≥ 60 years old and had a diagnosis of probable AD or probable DLB confirmed by two clinicians based on contemporaneous diagnostic criteria [19, 20], with an MMSE ≥ 12. Participants were recruited prior to the publication of the 2017 diagnostic criteria for DLB [1], but all DLB participants met the updated criteria for a diagnosis of probable DLB. Control participants were recruited through a research case register or were partners of participants.

Participants were excluded if they had a major concurrent psychiatric illness; severe physical illness; contraindications to PET-CT imaging or MRI; a history of other significant neurological illness including stroke or previous experimental treatment with an amyloid-targeting agent.

Participants with capacity gave their written informed consent to take part in the study. For those who lacked capacity to participate, their participation in the study was discussed with a consultee in accordance with the Mental Capacity Act. The study received ethical approval from the National Research Ethics Service Committee North East—Newcastle and North Tyneside 2 (13/NE/0064).

### Baseline cognitive and clinical assessment

Participants had a clinical and cognitive assessment carried out at baseline and 1 year. The severity of cognitive and functional impairment was assessed using the Addenbrooke’s Cognitive Examination-Revised (ACE-R) and the Bristol and Instrumental Activities of Daily Living Scales (BADL, IADL). IADL and BADL scores were combined to make a composite function z-score where positive scores indicated greater impairment. The severity of parkinsonism and cognitive fluctuations were measured using the Revised Unified Parkinson’s disease Rating Scale Motor Sub-scale (UPDRS) and Clinician assessment of Fluctuation (CAF), respectively. Blood pressure was assessed with the participant lying at rest and then after standing for 2 min. Comorbidities including vascular disease were assessed using the Cumulative illness Rating Scale for Geriatrics (CIRS-G) [21].

### Imaging

Imaging was performed at baseline. Details of the MRI and PET acquisition and analysis have been published elsewhere [22] and will briefly be summarised here.

Brain MRI scans were acquired using a 3T MR scanner (Achieva scanner; Philips Medical Systems), with body coil transmission and eight channel head coil receiver. Images acquired included a 3D sagittal magnetisation-prepared rapid gradient echo (MPRAGE) sequence (repetition time 8.3 ms, echo time 4.6 ms, flip angle 8°, inversion delay 1250 ms, imaging time 4.5 mins, sagittal acquisition matrix 216 × 240, voxel size 1 × 1x1mm) and T2*-weighted sequence (TR 1487 ms, TE 16.11 ms, flip angle 18°, 50 slices with 3 mm thickness (0 mm gap), voxel size 0.898 × 1.12 mm).

Amyloid imaging was carried out using a Siemens Biograph-40 PET-CT scanner with data acquired in list mode. Participants were given a 370 MBq intravenous injection of ^18^F-florbetapir (Amyvid), followed immediately by a 5 min scan for perfusion images, with a subsequent 15 min scan starting 30–50 min after injection to image amyloid distribution. Images were reconstructed using iterative reconstruction (4 iterations, 16 subsets), with a 168 × 168 matrix size, 2.04 × 2.04 mm pixel size, 3 mm slice thickness, and 3 mm post-reconstruction Gaussian filter. Attenuation correction was performed utilising CT scan data.

### Image processing

All analysis of MRI and PET images was performed using SPM 8 (www.fil.ion.ucl.ac.uk/spm/software/spm8/). MRI images were segmented into white matter, grey matter and CSF, and total intracranial volume was defined as the sum of these. White matter hyperintensity (WMH) segmentation was carried out using the FLAIR and T1 structural images using a semi-automated threshold-based algorithm employing SPM8 functions (www.fil.ion.ucl.ac.uk/spm) in an in-house written Matlab package (Matlab R2013a, Mathworks, Inc., Natick, MA, USA) as described previously [23]. The resulting WMH masks were manually edited using ITK-SNAP (www.itksnap.org) to correct for minor errors.

Grey matter volume (GMV) and white matter hyperintensities (WMH) were expressed relative to total intracranial volume. The log of WMH was used to normalise the data.

The amyloid PET image was co-registered with the native space MRI. A mean cortical standardised uptake value ratio (SUVR) was derived from the unweighted mean of frontal, temporal, parietal and cingulate regions relative to the whole cerebellum [24]. The perfusion region of interest SUVR was calculated from the early florbetapir images using a previously described method [22]. Hippocampal volumes were calculated using an automated technique [25].

The T2* images were processed to produce susceptibility weighted images (SWI) according to the method of Haacke using in-house Matlab software [26].

### Visual rating

The SWI images were rated by an experienced neuroradiologist (DM) who was blind to diagnosis and all clinical and other imaging measures. The Microbleed Anatomical Rating Scale [10] was used to rate their presence, number and location for each subject. Microbleeds were classed as definite or possible, and defined as present in a subject if there was any microbleed (definite or possible) in any region.

### Statistical analysis

Statistical analysis was completed using IBM SPSS Statistics software (version 22; https://www-03.ibm.com/software/products/en/spss-statistics). Demographic comparisons between microbleed groups were carried out using *t* tests or Mann–Whitney tests. *χ*^2^ or Fisher’s exact tests were used for categorical variables. The association of the presence of microbleeds with clinical and imaging variables was assessed using linear regression with age included as an independent variable. Associations were measured at baseline and follow-up. For follow-up measures, the dependent variable was rate of change of the measure, and baseline score was also included as an independent variable. Collinearity was excluded by ensuring correlation between independent variables was less than 0.7 and tolerance was greater than 0.1. The data were checked to ensure there were no outlying or influential values (standardised or studentised residual <  ± 3, leverage < 0.5, Cook’s Distance < 1). The normality of residuals was assessed by visual inspection of P–P plots of standardised residuals. Scatter plots of studentised residuals against predicted values and partial regression plots were inspected to ensure the presence of linear relationships between the dependent and independent variables and homoscedasticity in the overall model. Subjects with missing data were excluded from each analysis. Sample size was calculated to investigate amyloid imaging in DLB, AD and controls as previously reported [22].

## Results

A total of 87 participants agreed to enter the study, of which 72 met the eligibility criteria and underwent a baseline assessment. Two DLB cases were later excluded due to non-DLB pathological diagnosis; five DLB cases were confirmed by postmortem diagnosis. Two participants were unable to tolerate MRI. A total of 68 participants were included in this analysis (Table 1). DLB participants had significantly lower blood pressure than control participants. Both AD and DLB participants displayed predominantly lobar microbleeds.

**Table 1:** Demographics and microbleed results in DLB, AD and controls

Diagnosis	Control	AD	DLB	Statistic	*p*
*n*	20	18	30	–	–
Age	75.9 (7.3)	75.8 (7.1)	76.2 (7.0)	*F* < 0.1	0.98
Sex [*n*, (%) female]	4 (20%)	2 (11%)	7 (23%)	*χ*^2^ = 1.1	0.58
Smoking pack years	12.9 (21.5)	7.4 (15.2)	11.3 (20.8)	*H* = 0.2	0.88
Duration of dementia (months)	–	24.1 (21.8)	20.1 (19.4)	*U* = 242	0.54
On antiplatelet or warfarin [*n* (%)]	10 (50%)	3 (17%)	14 (47%)	*χ*^2^ = 5.5	0.06
On antihypertensive [*n* (%)]	12 (60%)	11 (61%)	14 (47%)	*χ*^2^ = 1.3	0.52
BP Lying systolic (mmHg)	160.5 (26.6)	141.2 (16.0)	143.4 (24.1)	*H* = 9.0	**0.01**^**b**^
BP Lying diastolic (mmHg)	80.2 (13.3)	73.9 (7.0)	73.2 (10.2)	*H* = 4.9	0.09
Definite MB lobar	0.1 (0.4)	1.3 (3.7)	0.3 (0.8)	*H* = 7.1	**0.03**^**a**^
Possible MB lobar	0.2 (0.5)	0.4 (1.0)	0.1 (0.3)	*H* = 1.5	0.47
Definite MB deep	0 (0)	0.1 (0.2)	0.0 (0.2)	*H* = 1.0	0.60
Possible MB deep	0 (0)	0 (0)	0.2 (1.1)	*H* = 1.3	0.53
Definite MB cerebellum	0 (0)	0.1 (0.2)	0.0 (0.2)	*H* = 1.0	0.60
Possible MB cerebellum	0 (0)	0 (0)	0.1 (0.3)	*H* = 2.6	0.28
Total MB	0.3 (0.9)	1.8 (4.6)	0.7 (1.3)	*H* = 6.1	**0.05**^**a**^
Any lobar MB [*n* (%)]	3 (15%)	9 (50%)	7 (23%)	*χ*^2^ = 6.3	**0.04**
Any deep MB [*n* (%)]	0 (0%)	1 (6%)	2 (7%)	*χ*^2^ = 1.3	0.51
Any cerebellar MB [*n* (%)]	0 (0%)	1 (6%)	3 (10%)	*χ*^2^ = 2.2	0.34
Any MB (n (%))	3 (15%)	9 (50%)	12 (40%)	*χ*^2^ = 5.6	0.06

Mean (SD) or *n* (%) where specified

Bold denotes *p* < 0.05. Bonferroni correction for post hoc comparisons

*MB* microbleed

^a^Significant control v AD

^b^Significant control v DLB

DLB participants with microbleeds tended towards being older and having a shorter disease duration than those without microbleeds and had higher resting systolic blood pressure (Table 2). There were no differences in smoking history, gender, vascular disease history, postural blood pressure change, years in education or rates of APOE-ɛ4 allele.

**Table 2:** DLB microbleed present and absent group demographics

	Microbleeds absent	Microbleeds present	Statistic	*p*
*n*	18	12	–	–
Age (years)	74.4 (8.1)	78.8 (3.9)	*t* = − 2.0	0.06
Sex [*n* (%) female]	4 (22)	3 (25)	FET	1
Duration of dementia (months)	25.8 (22.6)	10.4 (4.6)	*U* = 62	0.053
Smoking pack years	14.3 (24.7)	6.3 (11.2)	*U* = 87	0.61
Years in education	10.8 (2.2)	11.8 (5.1)	U = 83	0.31
APOE-ɛ4 allele present	11 (61)	6 (55)	FET	1
CIRS-G heart score	0.5 (1.0)	0.4 (0.8)	*U* = 108	1
CIRS-G vascular score	1.4 (1.1)	1.2 (1.0)	*U* = 96	0.60
CIRS-G total	11.2 (4.3)	11.8 (3.6)	*U* = 90	0.44
On antiplatelet or warfarin [*n* (%)]	8 (44)	6 (50)	*χ*^2^ = 0.1	0.77
On antihypertensive [*n* (%)]	9 (50)	5 (42)	*χ*^2^ = 0.2	0.65
On lipid medication [*n* (%)]	11 (61)	6 (50)	*χ*^2^ = 0.36	0.55
On cholinesterase inhibitor [*n* (%)]	16 (89)	11 (92)	FET	1
BP Lying Systolic (mmHg)	135.2 (19.0)	155.6 (26.4)	*U* = 55	**0.03**
BP Lying Diastolic (mmHg)	72.2 (10.5)	74.6 (10.1)	*t* = − 0.6	0.54
Postural systolic BP change (mmHg)	− 12.9 (16.8)	− 11.4 (32.9)	*t* = − 0.1	0.89
Postural diastolic BP change (mmHg)	− 0.5 (10.0)	− 1.4 (8.7)	*t* = 0.2	0.81

Mean (SD) or n (%) where specified

Significant findings in bold

*CIRS-G* Cumulative Illness Rating Scale-Geriatric, *FET* Fisher’s exact test

DLB participants with microbleeds had greater parietal perfusion (Table 3). There were no differences between the groups in grey matter volume, amyloid SUVR or white matter hyperintensities (Table 3). Similarly, those with specifically lobar microbleeds did not have a significantly higher amyloid SUVR (SUVR = 1.23 v. 1.25; Beta = − 0.12, *p* = 0.48).

**Table 3:** Imaging measures in DLB microbleed present and absent groups

	Microbleeds absent	Microbleeds present	Beta (95% CI)	*p*
*n*	18	12	–	–
Amyloid SUVR	1.25 (0.22)	1.25 (0.24)	− 0.17 (− 0.51 to 0.18)	0.33
Grey matter volume (% of ICV)	37.5 (3.2)	36.6 (2.1)	0.03 (− 0.23 to 0.27)	0.88
Hippocampal volume (% of ICV)	0.15 (0.04)	0.15 (0.02)	0.17 (− 0.11 to 0.46)	0.22
Log WMH	− 5.1 (1.2)	− 4.7 (0.6)	0.07 (− 0.28 to 0.42)	0.68
Parietal perfusion SUVR	0.83 (0.10)	0.93 (0.11)	0.42 (0.04–0.79)	**0.03**
Occipital perfusion SUVR	0.97 (0.07)	1.02 (0.06)	0.35 (− 0.05 to 0.74)	0.08
MTL perfusion SUVR	0.78 (0.10)	0.78 (0.07)	0.19 (− 0.15 to 0.52)	0.27

General linear model with age as a covariate. Microbleed present *n* = 12 for grey matter and hippocampal volume, otherwise *n* = 11

Significant findings in bold

*ICV* intracranial volume, *SUVR* standardised uptake value ratio, *WMH* white matter hyperintensities, *MTL* medial temporal lobe

DLB participants with microbleeds had better daily function scores and less severe parkinsonism (Table 4). When the duration of dementia was included as a covariate, the relationship with baseline function remained significant, whereas the relationships with baseline UPDRS (*p* = 0.08) and parietal perfusion (*p* = 0.07) were no longer significant.

**Table 4:** Baseline cognitive and clinical measures in DLB microbleed present and absent groups

	Microbleeds absent	Microbleeds present	Beta (95% CI)	*p*
*n*	18	12	–	–
ACE total	60.1 (15.3)	65.3 (18.8)	0.33 (− 0.03 to 0.68)	0.07
Function z-score	0.19 (1.10)	− 0.35 (0.76)	− 0.45 (− 0.78 to − 0.11)	**0.01**
CAF	7.6 (4.3)	4.8 (4.2)	− 0.22 (− 0.60 to 0.16)	0.24
MDS-UPDRS	47.9 (17.5)	33.8 (18.0)	− 0.40 (− 0.79 to − 0.02)	**0.04**

Mean (SD) or percentage where specified. General linear model with age as a covariate

Significant findings in bold

*ACE* Addenbrooke’s Cognitive Examination, *CAF* Clinician Assessment of Fluctuation, *MDS-UPDRS* revised Unified Parkinson’s Disease Rating Scale motor sub-scale

23/30 DLB participants completed the 1-year follow-up. Of those that did not undertake follow-up assessment, four had died, one had a severe stroke and two withdrew from the study. Those that did not complete follow-up tended towards being older and having more severe cognitive impairment. Four had microbleeds and three did not. One participant was unable to complete the ACE and another was unable to complete the UPDRS at follow-up. Participants with microbleeds had significantly less progression in parkinsonism measured by the UPDRS, though there was little change in the score in either group (Supplementary Table 1).

### Assumption checking for linear regression

There was a single significant outlier in baseline function and parietal perfusion analyses, but in both cases removing this subject had no effect on the result.

## Discussion

The aim of this study was to compare the rates of microbleeds in DLB with those in AD and healthy older people, and to compare the clinical and imaging findings of DLB with and without microbleeds. The prevalence of microbleeds in DLB was intermediate between controls and Alzheimer’s disease and was not statistically significantly different to either group. The lobar pattern of microbleeds in AD and DLB was in keeping with previous reports [15, 17, 27, 28], though one study has reported more deep microbleeds in DLB [16].

This is the first study to report both amyloid imaging and microbleeds in DLB and there was no evidence of increased amyloid deposition in the DLB participants with microbleeds. Our findings are in keeping with those from a small postmortem DLB cohort, which reported no increase in microbleeds in brains of people with DLB and concomitant Alzheimer’s disease or cerebral amyloid angiopathy [18]. However, these findings contrast with the established link between amyloid deposition and microbleeds in healthy older people [12, 13]. This association may have a significant interaction with age—with amyloid deposition being a greater risk factor for microbleed formation in younger ages [13]. The amyloid positive group in our study (SUVR > 1.11) had a mean age of 78.4 years, which may have been a factor in the absence of any association between amyloid SUVR and microbleeds.

This study found higher blood pressure in the group with microbleeds. This remained significant when age and disease duration were included as covariates (data not shown). Hypertension has been reported as a risk factor for deep, but not lobar, microbleeds in the Rotterdam Study [11]. However, the Mayo Study of Aging reported hypertension to be associated with lobar microbleeds, in keeping with our findings [13].

In this study, microbleeds in DLB were predominantly lobar. It is unclear if the mechanisms for the development of lobar microbleeds are different in healthy older people and those with DLB. This is plausible, as people with DLB have widespread cortical neurodegeneration, which could predispose to the development of microbleeds. De Reuck and colleagues [18] have suggested that microbleeds may occur in regions of increased angiogenesis related to pathology such as Lewy body disease. CSF markers of angiogenesis are known to be raised in Parkinson’s disease dementia [29], which is pathologically indistinguishable from DLB. It is possible that cortical vascular changes in DLB may make cortical vessels more susceptible to microbleeds. In such conditions, higher systolic blood pressure may be associated with a higher risk of microbleeds. Further pathological and imaging studies are required to test this hypothesis.

DLB participants with microbleeds had less functional impairment, less severe parkinsonism and higher parietal perfusion compared to those without microbleeds. However, the strength of these associations was attenuated when disease duration was included as a covariate and only the association with functional impairment remained significant. DLB participants with microbleeds had less progression in parkinsonism over 1 year. However, there was little difference between the two groups and the significance seems driven by differences in baseline score. It is difficult to draw conclusions from this clinical data due to the differences in disease duration; therefore, we think these findings should be interpreted with caution. One possible explanation is that vascular pathology has additive effects to Lewy body pathology in DLB. An additive effect of vascular disease and amyloid pathology on cognitive decline in healthy elderly people has been reported [30]. In addition, vascular pathology is an independent predictor of dementia in AD and synucleinopathy [5]. As such, vascular disease may result in the development of dementia in the presence of less advanced Lewy body pathology than would otherwise be expected in DLB. In such circumstances, Lewy body symptoms such as parkinsonism may also be less advanced. In the absence of an α-synuclein imaging biomarker, this hypothesis is difficult to test in vivo.

### Strengths and limitations

This study represents the most thorough assessment to date of the relationship of microbleeds with clinical and imaging measures in DLB. It is also the only study to report cortical amyloid SUVR in conjunction with cerebral microbleeds in DLB, providing evidence against the hypothesis that microbleeds in DLB are associated with amyloid pathology. Participants were recruited from a range of clinical services and have previously been demonstrated to have patterns of cognitive impairment and rates of amyloid deposition in keeping with the literature. These findings are therefore generalisable to clinical populations. Confirmatory diagnostic biomarkers were not assessed as part of this study, but were used to aid diagnosis where available.

The prevalence of microbleeds in AD was higher than in a recent meta-analysis. This most likely reflects the greater sensitivity of 3T susceptibility-weighted MRI used in this study, compared with lower field strengths in previous studies [14]. The number of microbleeds present in DLB cases was low, and unlikely to be sufficient to be the direct cause of clinical differences, but ample evidence shows that the number of microbleeds reported on MRI, even at 3T, underestimates the actual numbers present [31]. We compared DLB with possible or definite microbleeds to those with no microbleeds, as defined by Microbleeds Anatomical Rating Scale. We therefore cannot exclude the possibility that some of these microbleeds may have been mimics, e.g. calcification or iron deposition.

Due to the relatively low number of microbleeds it was not possible to compare amyloid deposition and microbleeds in different cortical regions. However, amyloid deposition in all regions correlated closely with the mean cortical SUVR (r > 0.90 in all areas).

The control group in this study had higher BP than AD or DLB. The reasons for this are unclear, though it may reflect more intensive medical management of people with dementia than healthy older people. Despite this, no healthy control had deep microbleeds, for which hypertension is a risk factor.

This was an exploratory analysis and therefore statistical correction for multiple comparisons across the manuscript was not made. The results require validation in other cohorts.

## Conclusions

Microbleeds in DLB are associated with higher blood pressure, but are not associated with other measures of vascular disease or amyloid deposition. The clinical significance of microbleeds in DLB remains uncertain.

## Electronic supplementary material

Below is the link to the electronic supplementary material.

Supplementary file1 (DOCX 12 kb)
