# Supplementary material for: Microbleeds in dementia with Lewy bodies
Source: J Neurol. 2020 Feb 4;267(5):1491–8. doi: 10.1007/s00415-020-09736-0 (PMC7184053; doi:10.1007/s00415-020-09736-0)
Supplement: Supplementary file 1 — Supplementary file1 (DOCX 12 kb) [file 415_2020_9736_MOESM1_ESM.docx]

| **Supplementary Table 1.** Change in cognitive and clinical scales over one year in DLB microbleed present and absent groups | | | | |
| --- | --- | --- | --- | --- |
|  | Microbleeds absent | Microbleeds present | Beta (95% CI) | p |
| n | 15 | 8 | - | - |
| ACE Total | -6.6 (10.2) | -6.6 (10.2) | 0.35 (-0.12-0.83) | 0.14 |
| Z score function | 0.08 (0.76) | -0.26 (0.61) | -0.43 (-0.98-0.11) | 0.11 |
| CAF | 1.0 (4.8) | 2.2 (3.0) | -0.23 (-0.56-0.10) | 0.17 |
| MDS-UPDRS | 0.1 (8.8) | -1.9 (9.6) | -0.55 (-0.94- -0.17) | **0.01** |
| Mean (SD). General linear model with age and baseline score as a covariates. ACE-Addenbrooke’s Cognitive Examination; MDS-UPDRS-revised Unified Parkinson’s Disease Rating Scale motor sub-scale. Microbleed absent n=14 for ACE Total and MDS-UPDRS. Significant findings in bold. | | | | |
